# Preventing comorbidity between distress and suicidality: a network analysis

**DOI:** 10.1038/s44184-023-00022-1

**Published:** 2023-03-04

**Authors:** Alvin Junus, Paul S. F. Yip

**Affiliations:** 1 Department of Social Work and Social Administration, The University of Hong Kong, Hong Kong SAR, China; 2 The Hong Kong Jockey Club Centre for Suicide Research and Prevention, The University of Hong Kong, Hong Kong SAR, China; 3 HKU Institute of Data Science, The University of Hong Kong, Hong Kong SAR, China

**Keywords:** Psychiatric disorders, Risk factors, Public health, Social sciences

## Abstract

Suicidality among individuals between 10 and 35 years of age may be poised to exert massive burdens on society through decreased economic productivity and increased incidence of chronic physical conditions in the individuals’ later years, thereby necessitating early prevention of suicide. While research suggests that the pathway to suicidality may begin from episodes of psychological distress, such pathway may involve complex interplays between intermediary psychiatric symptoms and external stimuli that are not easily delineated through conventional means. This study applies the network approach to psychopathology to elucidate this complexity. Comorbidity between psychological distress and suicidality in 1968 community-dwelling individuals is analyzed with regularized partial correlation networks to identify their bridge symptoms and links. Temporal relationships between symptoms are analyzed through temporal symptom network formed from 453 individuals who completed subsequent follow-up surveys. Network analysis shows that feelings of hopelessness and the presence of suicidal ideation are the strongest bridge symptoms in the comorbidity symptom network, and form the only prominent link that bridges psychological distress and suicidality. Effects of sleep troubles, anxiety, and poor social relationships on suicidal ideation appear to be mediated by hopelessness. The same observations hold among individuals with and without diagnoses of psychiatric disorders, as well as young people (10–24 year-olds) and young adults (25–35 year-olds). The edge between hopelessness and suicidal ideation remains the strongest bridge link after controlling for effects of symptoms from the previous time point. Findings here provide an evidence base for both professional training in caregiving professions as well as gatekeeper training in community members to emphasize more on how to effectively recognize hopelessness, and instill hope, in young people and young adults for various types of distress.

## Introduction

Mental ill-health among individuals in their second and third decades of life has been a pressing public health challenge worldwide in recent years, even before the Covid-19 pandemic struck. The ages of 15 and 30 are peaks for the onset of mental disorders^1^, among which suicidality is particularly urgent. Suicide is now the fourth leading cause of death among youths globally^2^. Likewise, suicide has consistently been the leading cause of death for individuals between 10 and 35 years of age in societies such as Hong Kong^3,4^, although precipitating factors might differ across developmental stages, i.e., suicide among young people (10–24 year-olds^5^) and young adults (25–35 year-olds^6^) was mainly precipitated by school-related events and financial issues respectively^7^. Moreover, suicide among these demographic groups is still persistently on the rise in recent years^8^. Thus, suicide may be poised to exert massive burdens on society in the coming years in the form of decreased economic productivity of^9^, as well as increased incidence of chronic physical conditions in, the suicidal individuals’ later years^10^. Early detection of suicidality for these demographic groups, and subsequently its early prevention, are therefore imperative in order to avert this looming societal crisis^11^.

Suicidality may be classified into three stages—ideation, planning, and attempt—with each successive stage more severe than and theorized to be causally progressing from its preceding stage^12^. Research has suggested that the pathway to suicidality in an individual, particularly suicidal ideation, may begin from episodes of psychological distress or pain such as those triggered by stressful life events^13–15^. Studies have long observed that prolonged psychological distress may lead to conditions such as depression, anxiety, feelings of hopelessness, lack of sleep, and poor interpersonal relationships^12,16,17^, which are themselves well-established risk factors of suicidality^18–22^. However, the pathway from distress to suicidality necessarily involves complex interplays between these conditions/symptoms and external stimuli, which may include feedback loops and mutually reinforcing activations of symptoms, among others. Such interactions are not easily delineated through conventional means, thereby hindering scientific advances in early intervention of suicidality^14,19^.

The network approach to psychopathology^23^ has much potential in elucidating this complex pathway. Under this approach, a mental disorder emerges from mutual interactions and often reciprocally reinforcing activations of its constituent symptoms. Patterns of symptom-symptom interactions are encoded in a network structure. Symptoms are conceptualized as *nodes* in a network, and a connection / an *edge* between two symptoms implies that the activation of one symptom directly leads to the activation of the other. In this respect, a particular mental disorder is equivalent to a *community* of symptoms, i.e., a cluster of symptoms that are strongly connected to one another^24^. For example, an individual’s suicidality can be characterized by symptoms such as suicidal thoughts, feelings of torment, control over self-harming thoughts, and attempts at suicide, all of which can causally interact with and reciprocally reinforce one another.

Following this paradigm, comorbidity between distress and suicidality would be a natural consequence of overlapping connectivity between the two disorders’ communities of symptoms^25^. Certain *bridge symptoms* in one community would have edges to symptoms in the other community, and the activation of such bridge symptoms may in turn activate the connected symptom in the other community, thus giving rise to comorbidity between the two disorders. Specifically, such bridge symptoms in the distressed community may have edges to an early symptom of suicidality, i.e., suicidal ideation; the progression from suicidal ideation into the more severe symptoms of suicide planning or attempt^12^ would be encoded as edges within the suicidality community. The activation of these bridge symptoms during a prolonged episode of distress may then activate suicidal ideation, resulting in the distressed individual becoming suicidal.

Understanding which symptoms bridge distress and suicidality among individuals between 10 and 35 years of age can offer key insights into the early intervention of suicidality tailored for this demographic group. Early treatment of these bridge symptoms in a distressed individual may lead to the pathways linking the two communities being pre-emptively deactivated, and potentially prevent their distress to progress into suicidality. When translated to a larger scale, e.g., through screening and training programs, such initiatives can potentially reduce the incidence of suicidality at the population level. The central research question addressed in the present study is therefore: *What are the symptoms giving rise to comorbidity between distress and suicidality among individuals aged 10 to 35 years?*

Following the network approach to psychopathology, this study estimates a regularized partial correlation network consisting of distress and suicidality symptoms using longitudinal online survey data of community-dwelling individuals aged 10 to 35 years in Hong Kong. The network structure is analyzed to identify bridge symptoms linking distress to suicidality. Further analyses examine whether the same bridge symptoms and edges between them significantly differ in specific subpopulations after controlling for temporal relationships between symptoms.

## Methods

### Participants and procedures

Data for this study were obtained from longitudinal online surveys that were conducted annually from 2018 to 2020. The surveys asked questions on general well-being and suicidality, and this initiative was led by the Centre for Suicide Research and Prevention (CSRP) at the University of Hong Kong (HKU). Targeted study samples were 10–35 year-old individuals living in the general Hong Kong population, particularly those with known risk factors of suicide such as previous suicidal ideation and attempt, and psychiatric disorders.

For the first survey wave, links to the online survey were disseminated through poster promotions, emails to members, newsletters, and Facebook and web pages of the authors’ affiliated institutions. Additionally, for maximum outreach to the targeted participants, links to the survey were also disseminated as (i) poster promotions at branches, and (ii) notices to members of, three community outreach organizations: Caritas, Hong Kong Federation of Youth Groups, and The Boys’ and Girls’ Clubs Association of Hong Kong. All three are major outreach organizations with territory-wide service centers that provide counseling and social work services to individuals up to 35 years of age^26–28^.

Clicking the survey link would direct participants to a secure webpage containing the survey. Participants could choose to fill out either a Chinese or English version. Written informed consent was first obtained from all participants, and they were informed of the survey’s purpose (gaining an in-depth understanding of their demographic group’s general well-being), approximate survey duration (ten minutes), strict confidentiality of their data, and of their freedom to discontinue at any time. Careful consideration was taken to ensure that the survey questions would incur no risk and pose the least stress to participants. Contact information for emotional support hotlines and services was made available throughout the survey to encourage distressed participants to seek support immediately. Participants who consented to be contacted further for follow-up could provide their email addresses, where survey links were sent in the subsequent survey collection period.

All procedures & protocols adopted in this study were approved by the Human Research Ethics Committee for Non-Clinical Faculties of HKU under the reference number EA1709039. Consent from parents or legal guardians for under-aged participants was deemed not required by the committee as the endorsed study was assessed to pose minimal potential harm to under-aged participants. Survey collection periods for 2018, 2019, and 2020 waves were 22 December 2017–15 July 2018, 5 June–8 July 2019, and 29 June–29 September 2020 respectively. In 2019 and 2020 survey waves were follow-up surveys from the previous wave, and thus did not involve any recruitment of new participants.

### Measures

#### Demographics and risk factors of suicidality

Participants’ basic demographics of interest were age and gender. The baseline (2018) survey further inquired participants’ experiences on known risk factors of suicidality: Whether they had ever considered suicide, self-harmed, and attempted suicide in their lifetime; and whether they had been diagnosed with psychiatric disorders such as major depressive disorder (MDD) and schizophrenia. All questions on risk factors of suicidality were binary-response (“yes” and “no”) questions.

#### Chinese health questionnaire (CHQ-12)

Distress was measured by the twelve-item Chinese Health Questionnaire (CHQ-12)^29^, which has seen wide usage in assessing overall psychological health and screening for psychological distress symptoms among Chinese community-dwelling populations^30,31^. Validations of CHQ-12 in multiple settings have shown specificity and sensitivity values between 0.70 and 0.95^30,32^, and Cronbach’s alpha higher than 0.83^33,34^, indicating good internal consistency. CHQ-12 assesses the severity of physical, social, and emotional distress experienced by an individual in the past two weeks. Each item is scored on a 4-point Likert scale from 0 to 3, with higher value indicating higher severity. Two items are reverse-scored: (i) feeling that one is getting along with family and friends; and (ii) feeling hopeful about the future. Supplementary Table 1 shows the CHQ-12 used in the surveys. Cronbach’s alpha for CHQ-12 in the present study was 0.85.

#### Suicidal ideation attributes scale (SIDAS)

Study participants’ suicidality was measured by the five-item suicidal ideation attributes scale (SIDAS)^35^. SIDAS is designed to screen individuals in the community for the presence of suicidal thoughts in the past month and to assess the severity of these thoughts, e.g., whether they progressed into a suicide attempt. It measures the frequency and uncontrollability of suicidal thoughts, closeness to suicide attempt, and distress and interference brought by the thoughts. Supplementary Table 2 shows the SIDAS used in the present study. Each item is scored on an 11-point Likert scale from 0 to 10, with higher value indicating higher severity. A score of 10 on the third item corresponds to at least one suicide attempt in the past month. A total score of 0 indicates no suicidality within the past month. Participants who gave a response of 0 to the first item, i.e., never having any suicidal thoughts in the past month, automatically skipped all remaining items and scored a total of 0. Validation of SIDAS within the Chinese context has shown convergent validity and Cronbach’s alpha of 0.84, indicating good internal consistency^36^. Cronbach’s alpha for SIDAS in this study was 0.91.

### Data analysis

All analyses in this study were conducted on R version 4.2.1^37^ within RStudio^38^, using the packages *qgraph*^39^, *bootnet*^40^, *networktools*^41^, *psychonetrics*^42^, and *NetworkComparisonTest*^43^. There was no missing data in the study population. The main analyses of this study focused on responses in the 2018 survey wave, whereas the three-year panel data based on responses of individuals who completed all three surveys (2018 to 2020) were utilized in additional analyses that investigated temporal relationships between symptoms.

#### Network structure estimation and visualization

Each CHQ-12 and SIDAS item was conceptualized as a node. Two communities were then defined: A distress community consisting of the 12 symptoms measured by CHQ-12 and a suicidality community consisting of 5 suicidality symptoms measured by SIDAS. Altogether, a distress-suicidality symptom network consisting of 17 nodes was conceptualized.

To obtain the symptom network’s structure, edges between all possible pairs of nodes were estimated using regularized *partial correlation*. For every pair of symptoms, Spearman correlation was calculated after controlling for all other associations with all other symptoms in the network. The strength of the edge between two nodes, i.e., *edge weight*, thus denoted the partial Spearman correlation coefficient between the two nodes. The graphical least absolute shrinkage and selection operator (gLASSO) algorithm^44^ was used such that small partial correlations that were likely due to noise were set to exactly zero, thereby resulting in a sparse network. Model selection on the regularized network was then conducted using the extended Bayesian Information Criterion (EBIC) with a conservative tuning hyperparameter *γ* = 0.5 in order to obtain a network with high specificity^45^.

Separately, graphical vector autoregression (GVAR)^46^ was employed on the three-year panel data to elucidate temporal dependencies between symptoms and disentangle them from relationships between symptoms within the same window of measurement. This was implemented using the *psychonetrics*^42^ package. Three network structures were estimated based on the variance-covariance structure of the data: (i) A lag-1 *temporal network* that encoded partial correlations between symptoms in one panel and those in the next; (ii) a *contemporaneous network* which encoded expected partial correlations between symptoms within a panel after having controlled for effects of symptoms in the previous panel; and (iii) a *between-subjects network* which encoded within-panel relationships between stationary means of different individuals. Contemporaneous and between-subject networks were modeled as Gaussian graphical models.

A model in which all edges were included was first fit using maximum likelihood estimation. Edges that were not significant at significance level *α* were set to zero, the model was refit to yield a *pruned* model, followed by a stepwise-up model search strategy at the 0.05 significance level, and model-fit statistics of the resulting model—RMSEA, CFI, TLI—were then evaluated. The procedure was repeated for decreasing *α*, and the eventual model with the lowest *α* that still yielded RMSEA < 0.08, CFI > 0.90, and TLI > 0.90 was selected.

Visualization of the estimated networks was done with the Fruchtermann-Reingold algorithm^47^. While the algorithm facilitates readability, it does not provide a meaningful interpretation of edges’ lengths. Edge thickness reflected the magnitude of correlations, i.e., the aforementioned edge weight, with thicker edges indicating higher edge weight. For a temporal network, directed edges between nodes represented partial directed correlations between symptoms over time – an edge from one node to another denoted a partial correlation between the former to the latter at the next time point after controlling for the effects of all other nodes at the first time point.

#### Centrality indices

*One-step expected influence (EI)* and *one-step bridge EI* were the primary centrality indices used to identify important individual and bridge symptoms in the estimated networks^48,49^ respectively. We note that while other centrality indices such as degree, betweenness, and closeness centrality have been commonly adopted in prior studies in network psychopathology, those measures are conceptually less relevant to a weighted symptom network^50,51^ and are thus not relied on in this study. We also note that EI is preferred to strength centrality here since both indices would be equivalent in a network with only positive edges, while EI centrality has been suggested to be better than strength centrality at assessing nodes’ influence in a network with negative edges^48^.

A node’s one-step EI is the sum of all edge weights directly connecting it to other nodes. For node *i* in a network with *N* nodes,

1 $$\,{{\mbox{Node}}}\,\,E{I}_{i}=\mathop{\sum }\limits_{j=1}^{N}{w}_{ij},$$

where *w*_*ij*_ denotes the weight of the edge connecting nodes *i* and *j*. *w*_*ij*_ = 0 if no edge exists.

One-step bridge EI is an extension of the same notion to assess a node’s importance in comorbidity between disorders. Given a network with predefined communities, each of which represents a distinct disorder, a node’s one-step bridge EI is the sum of all edge weights directly connecting it to nodes in other communities, thereby indicating the node’s total connectivity with other disorders. For node *i* belonging to community *C* in a network with *N* nodes,

2 $$\,{{\mbox{Bridge}}}\,\,E{I}_{i}=\mathop{\sum}\limits_{j\notin C}{w}_{ij},$$

where *w*_*ij*_ denotes the weight of the edge connecting nodes *i* and *j*. *w*_*ij*_ = 0 if no edge exists. For both indices, higher value for a node indicates that the node holds a higher centrality in the network; activation of a node with higher EI and bridge EI should have greater influence on, or higher probability of, activation of other nodes and onset of a comorbid disorder respectively.

#### Network accuracy, stability, and comparisons

Before making any inference on the estimated networks, bootstrapping procedures were employed to assess the accuracy of edge weights and stability of centrality indices^40^. Specifically, for each estimated network, 1000 bootstraps were conducted to obtain a 95% confidence interval (CI) of all edge weights in the network. A narrower interval would indicate a more accurate edge weight estimate. *Bootstrapped difference test* between two edges, i.e., taking the difference between bootstrap values of one edge weight and another edge weight, and constructing a bootstrapped CI around those difference scores^40^, was further conducted to compare specific edges of interest. The absence of zero in the bootstrapped CI would indicate that the two edges’ weights significantly differed from each other.

The stability of centrality indices was evaluated through *case-dropping subset bootstrap*, where various proportions of cases/individuals were randomly dropped from the full sample, and networks were re-estimated from the resulting subsets. 1000 bootstraps were conducted. For each bootstrap, centrality indices obtained from the subsets were compared with their original indices using the *correlation stability coefficient* (CS-coefficient), which denotes the maximum proportion of cases that can be dropped, such that with 95% probability the correlation between the original centrality indices and those based on subsets is at least 0.70. CS-coefficients from each bootstrap were then averaged. A high average CS-coefficient indicates high stability of the centrality indices. For reliable interpretation, a centrality index’s average CS-coefficient should not be below 0.25, and preferably above 0.50^40^.

Additionally, networks based on specific subpopulations were estimated and evaluated for sensitivity analyses. This was to investigate whether bridge symptoms and links identified in the full-sample network differed for distinct subpopulations and to also assess differences between networks resulting from complementary subpopulations. A network was estimated from individuals who had been diagnosed with psychiatric disorders such as MDD or schizophrenia, and was compared with one estimated from individuals who had not. Likewise, separate networks were estimated for young people (10–24-year-old individuals) only and young adults (25–35-year-old individuals) only and then compared.

Network comparisons were performed using the package *NetworkComparisonTest*^43^, which defined test statistics used for comparing a pair of networks. For a pair of networks 1 and 2,

3 $$M=\max \left\vert{w} _{ij}^{1}-{w}_{ij}^{2}\right\vert \quad \forall \{i,j\},$$

4 $$S=\mathop{\sum}\limits_{ij}\left\vert {w}_{ij}^{1}\right\vert -\mathop{\sum}\limits_{ij}\left\vert {w}_{ij}^{2}\right\vert \quad \forall \{i,j\},$$

where ${w}_{ij}^{1}$ and ${w}_{ij}^{2}$ denote the weight of the edge connecting nodes *i* and *j* in network 1 and 2 respectively. Two hypothesis tests were conducted for each comparison between a pair of networks: (i) an omnibus *network invariance test*, where the null hypothesis stated that all corresponding edges in both networks were equal, i.e., *H*_0_ : *M* = 0; and (ii) *global strength invariance test*, where the null hypothesis stated that both networks had the same overall connectivity, i.e., *H*_0_ : *S* = 0. Test statistics calculated from the estimated networks were compared with distributions of their corresponding values generated from 1000 permutations under the null hypothesis (see ref. ^43^). For each test, *p* > 0.05 would indicate that the test could not identify differences in (i) corresponding edges’ strengths and (ii) overall connectivity of the two networks respectively, and thus the null hypothesis would be accepted.

### Reporting summary

Further information on research design is available in the Nature Research Reporting Summary linked to this article.

## Results

### Sample characteristics

There were 1968 individuals who completed the baseline survey wave in 2018, and among them, 453 individuals completed all three surveys. Table 1 summarizes the descriptive statistics of the study population in the 2018 survey wave. The study population’s mean age (standard deviation) was 22.3 (5.2) years. Females constituted around two-thirds of the study population. Almost one-tenth of the study population had been diagnosed with either MDD or schizophrenia. Approximately one-third had suicidal ideation in the past month, and there was also a high lifetime prevalence of suicidal ideation, self-harm behaviors, and suicide attempt. Supplementary Fig. 1 shows distributions of CHQ and SIDAS responses. No statistically significant difference between the full (*N* = 1968) and panel (*N* = 453) samples was observed for all measured variables.

**Table 1:** Descriptive statistics of the study population in the baseline 2018 survey wave.

Gender	*n* (%)
Female	1353 (68.8%)
Male	615 (31.2%)
Age group	
10–14	78 (4.0%)
15–19	623 (31.7%)
20–24	654 (33.2%)
25–29	399 (20.3%)
30–35	214 (10.9%)
Risk factors of suicidality	
Lifetime prevalence of suicidal ideation	890 (45.2%)
Lifetime prevalence of self-harm	519 (26.4%)
Lifetime prevalence of suicide attempt	154 (7.8%)
Psychiatric disorder diagnosis	195 (9.9%)
CHQ-12 total score [mean (s.d.)]	13.7 (6.6)
SIDAS score [mean (s.d.)]	5.1 (9.3)
0	1248 (63.4%)
≥1	720 (36.6%)

Values are expressed as [*n* (% of study population)] unless indicated otherwise. Numbers are rounded to 1 decimal place.

### Symptom networks

**Fig. 1:**
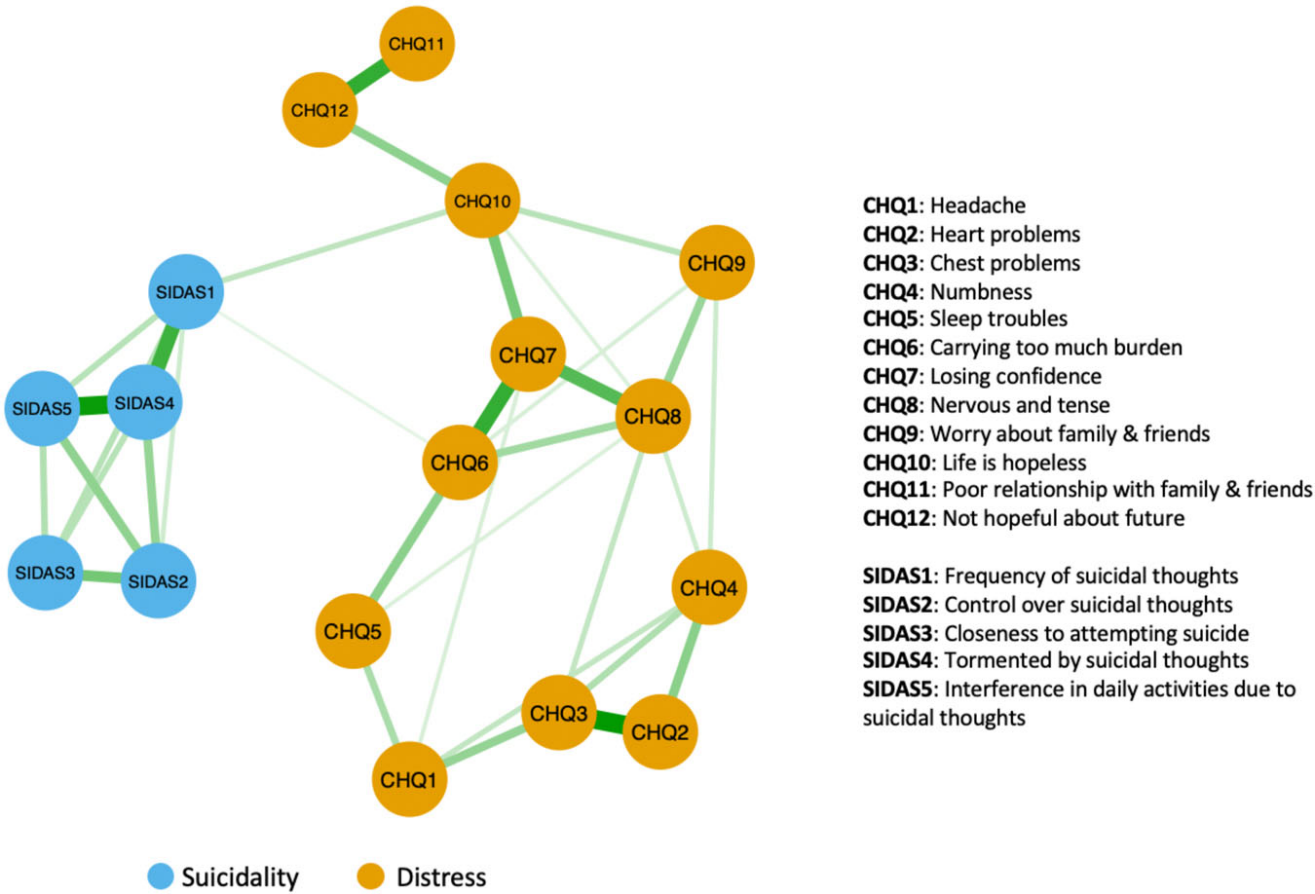
Distress-suicidality symptom network constructed from responses of 1968 individuals in the baseline 2018 survey. Edge thickness represents the magnitude of the partial correlation between symptoms. Blue nodes and orange nodes represent suicidality and distress symptoms respectively. All edge weights are positive.

Figure 1 illustrates the full-sample symptom network. All identified edges were positive. Edge weights of the network were within their corresponding bootstrap estimates (see Supplementary Fig. 2), implying that the current network structure was stable. While most edges were intra-community edges, there were two edges connecting the two communities: The CHQ10 – SIDAS1 (hopelessness – suicidal ideation) edge and the CHQ6 – SIDAS1 (burden – suicidal ideation) edge. The weight of CHQ10 – SIDAS1 in the network was 0.12, higher than 0.05 for CHQ6 – SIDAS1. Bootstrapped mean [95% CI] of the weight of CHQ10 – SIDAS1 was 0.12 [0.08,0.16], whereas that of CHQ6 – SIDAS1 was 0.03 [−0.01,0.11]. Bootstrapped difference test on the two edges yielded a bootstrapped 95% CI of [0.01,0.15], signifying that the CHQ10 – SIDAS1 was a substantially stronger edge that linked the distress and suicidality communities.

**Fig. 2:**
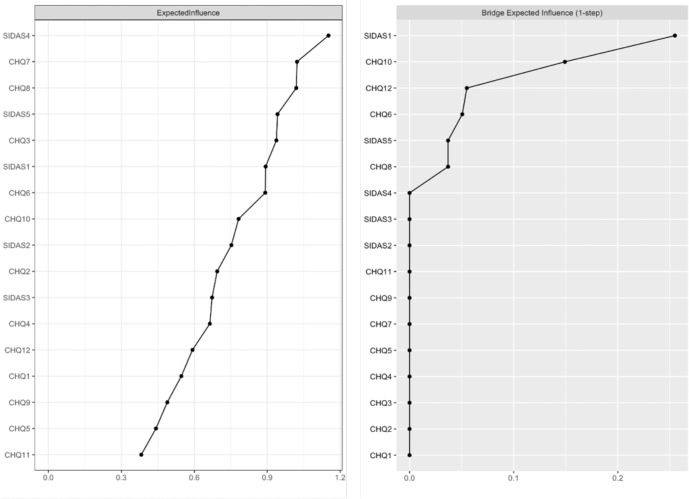
Values of centrality indices in the full-sample symptom network. CHQ1 Headache, CHQ2 Heart problems, CHQ3 Chest problems, CHQ4 Numbness, CHQ5 Sleep troubles, CHQ6 Carrying too much burden, CHQ7 Losing confidence, CHQ8 Nervous and tense, CHQ9 Worry about family & friends, CHQ10 Life is hopeless, CHQ11 Poor relationship with family & friends, CHQ12 Not hopeful about future, SIDAS1 Frequency of suicidal thoughts, SIDAS2 Control over suicidal thoughts, SIDAS3 Closeness to attempting suicide, SIDAS4 Tormented by suicidal thoughts, SIDAS5 Interference in daily activities due to suicidal thoughts.

Node EI and bridge EI CS-coefficients were 0.75 and 0.52 respectively (see Supplementary Fig. 3, indicating that both centrality indices were robust and could be reliably interpreted. Figure 2 shows node EI and bridge EI values of each node in the full-sample symptom network. SIDAS4 (feelings of torment from suicidal ideation), CHQ7 (loss of confidence), and CHQ8 (nervousness) had the highest values for node EI, which implied that their activation would have the highest probabilities of causing direct activation of their connected symptoms, e.g., activation of CHQ7 leading to activation of CHQ6, CHQ8, and CHQ10. On the other hand, SIDAS1 (presence of suicidal ideation) and CHQ10 (feeling of hopelessness) were by far the strongest bridge symptoms in the network, as exemplified by their bridge EI values. In addition, there was also an absence of edges between SIDAS1 and nodes such as CHQ5 (sleep troubles), CHQ8 (anxiety), and CHQ11 (poor social relationships), which are well-established risk factors of suicidality. These nodes were instead indirectly connected to SIDAS1 through CHQ10.

Supplementary Fig. 4 illustrates sub-sample networks for (i) 195 individuals who had been diagnosed with either MDD or schizophrenia and for the remaining 1773 individuals who had not, while Supplementary Fig. 5 shows sub-sample networks for (ii) 1355 young people (10–24-year-old individuals) and 613 young adults (25–35-year-old individuals). CS-coefficients for each sub-sample network indicated robust network centrality indices (see Supplementary Fig. 6). Similar in the full-sample network, both SIDAS1 and CHQ10 were still the strongest bridge symptoms in all sub-sample networks (see Supplementary Fig. 7). Similarly, the CHQ10 – SIDAS1 edge was the only bridge link between the distress and suicidality communities. Bootstrapped edge weight mean [95% CI] for the CHQ10 – SIDAS1 edge was 0.16 [0.08,0.30] and 0.12 [0.08,0.17] for the network based on individuals with and without diagnosis respectively, and 0.11 [0.06,0.17] and 0.10 [0.01,0.23] for the network based on young people and young adults respectively. Comparison tests found no significant difference between psychiatric diagnoses sub-sample networks (network invariance test: *M* = 0.19, *p* = 0.49, and global strength invariance test: *S* = 0.13, *p* = 0.75) and similarly, no significant difference between age group sub-sample networks (network invariance test: *M* = 0.14, *p* = 0.40, and global strength invariance test: *S* = 0.17, *p* = 0.43).

### Temporal relationships between symptoms

**Fig. 3:**
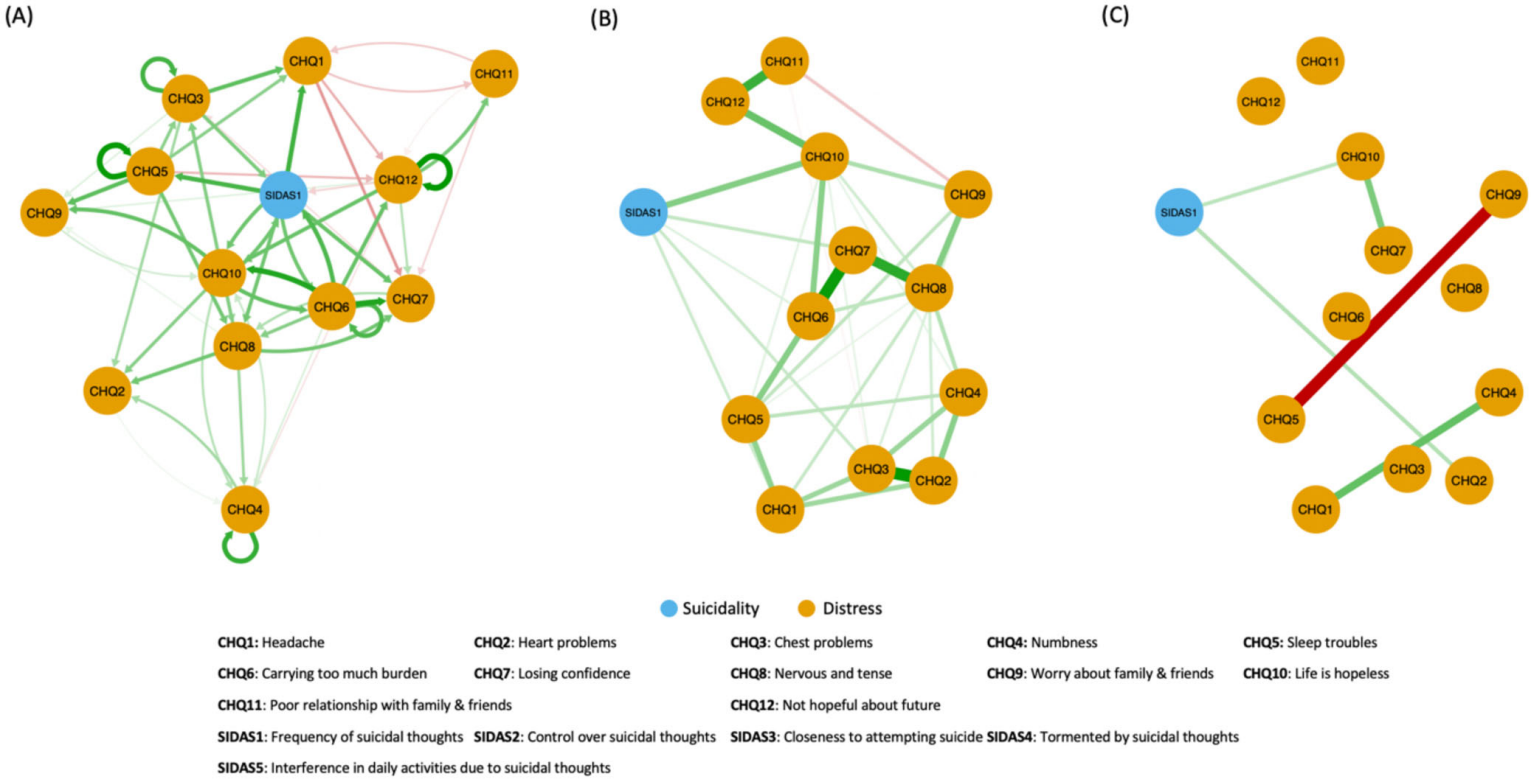
Temporal network of distress and suicidality symptoms constructed from responses of 453 individual over surveys in 2018, 2019, and 2020. Network structures of distress and suicidality symptoms constructed from responses of 453 individual over surveys in 2018, 2019, and 2020: (**A**) lag-1 temporal network, (**B**) contemporaneous network, and (**C**) between-subjects network. Green and red edges denote positive and negative correlations respectively. Edge thickness reflects the magnitude of correlation. Blue nodes and orange nodes represent suicidality and distress symptoms respectively.

Figure 3A–C shows the lag-1 temporal network, contemporaneous network, and between-subjects network based on responses of the 453 individuals who completed the surveys in all of 2018, 2019, and 2020. Among the SIDAS items, only SIDAS1 was included so as to avoid convergence issues during model estimation while still sufficiently investigating temporal effects on the CHQ10—SIDAS1 link. *α* of the model was 0.20. Model-fit statistics were TLI = 0.93, CFI = 0.93, and RMSEA = 0.042. Separate symptom networks for each panel were depicted in Supplementary Fig. 8.

Figure 3A showed, among others, bidirectional positive partial correlation between CHQ6 (carrying too much burden) and CHQ10 (feeling of hopelessness) and somewhat weaker bidirectional negative partial correlation between CHQ1 and CHQ11. There was notably no autocorrelation observed for SIDAS1. Importantly, the temporal network showed bidirectional positive partial correlation between SIDAS1 (presence of suicidal ideation) and CHQ10. This indicated that the presence of suicidal thoughts at one-time point was associated with hopelessness at the next time point and vice versa. The same relationship could be observed for SIDAS1 and CHQ6. Figure 3B, in particular, suggested that within-panel link between CHQ10 and SIDAS1 remained strong even after accounting for effects from the previous time point, as indicated by its thickness, thus reiterating findings from the baseline survey. Lastly, Fig. 3C showed that within the same panel, individuals with more frequent feelings of hopelessness also had more frequent suicidal ideation and heart problems compared to those with less hopelessness.

## Discussion

The present study drew upon the network approach to psychopathology in order to delineate the complex pathway from psychological distress to suicidality among community-dwelling individuals aged 10 to 35 years. This paradigm allows researchers to go beyond conventional moderation and mediation studies that typically involve only a few interactions, and with which a nuanced pathway has hitherto remained elusive to uncover^14,19^. In this regard, therefore, this paper represents a novel attempt to characterize the complex structure of interactions between psychiatric symptoms that would enable an episode of psychological distress to progress to suicidality for the studied demographic group. Findings here may provide broader insights into the early prevention of suicidality for distressed community-dwelling individuals of the same demographic group in other societies.

Gender ratio of the study population aligned with the established understanding that at-risk females are more inclined to seek community outreach organizations for help^52,53^. Analyses on distress-suicidality symptom networks revealed two bridge symptoms that were pivotal in giving rise to comorbidity between psychological distress and suicidality: (i) Feelings of hopelessness and (ii) the presence of suicidal ideation. In particular, these two symptoms formed the only prominent link that bridged the two conditions. The same observation was held for individuals with and without diagnoses of psychiatric disorders such as MDD and schizophrenia, and also for those below 25 years of age and older alike. These findings, therefore, suggested that loss of hope might be a key tipping point that differentiated between the presence or absence of suicidal ideation in a distressed young individual. This naturally lends further support for the recently proposed Three-step Theory of Suicide (3ST)^12^, specifically contributing to the growing body of empirical evidence corroborating its first step^54^, which posits that suicidal ideation results from a combination of (psychological) pain and hopelessness. More broadly, findings here also serve to further demonstrate the network perspective’s potency in elucidating intricate symptom-level interconnections between distinct disorders that could have been obscured otherwise had the disorders been operationalized as sum scores^51^.

While there is a prevailing understanding that symptoms such as sleep troubles, anxiety, and poor social relationships are associated with suicidal ideation^19–21^, the symptom networks’ structure here nevertheless illustrated that activations of these symptoms in a distressed individual were only indirectly correlated with activation of suicidal ideation; instead, their effect on suicidal ideation appeared to be mediated by hopelessness. On one hand, this finding echoed the same conclusions reached in extant studies^55,56^. Yet, each of these studies considered separate subsets of factors and thus only presented fragmented pictures of limited scope. With the same finding established after accounting for a much broader set of factors, this study, therefore, extends the literature by providing a richer and more complete picture of the pathway to suicidality. Concurrently, the prominent mediating role of hopelessness here could be indicative of Granger’s causality, subsequently suggesting that hopelessness might be a stronger precursor of suicidal ideation than the other aforementioned risk factors were. As such, signs of a distressed individual losing hope for the present should be taken as a warning signal to redouble support for the individual.

The absence of a self-reinforcing feedback loop for suicidal ideation in the temporal network suggests a more episodic nature of suicidal ideation among the study population, as was similarly observed among psychiatric inpatients^57–59^. On the other hand, it appeared that the presence of suicidal thoughts and hopelessness mutually reinforced each other over time in what seemed to be a perilous cycle. While this observation might correspond to at-risk individuals facing continued underlying problems, e.g., separated parents, family hardships, etc., the mismatch between the time gap between panels and survey measurements’ timeframe still warrants caution in interpreting this observation. Nevertheless, within a one-month cross-section, hopelessness remained the strongest link to the presence of suicidal ideation in a distressed individual. It thus follows that a community monitoring mechanism that is both sensitive to the potential for suicide and also capable of responding rapidly to possible onsets of suicidal ideation in community-dwelling individuals would be essential in realizing effective early intervention of suicidality at the population level.

Such a mechanism should, in light of the findings here, focus on timely detecting and deactivating hopelessness in distressed individuals. Professional training in social work and counseling professions should place more emphasis on how to effectively recognize hopelessness, and instill hope, in young people and young adults for various types of distress. A similar module should also be extended to professional development programs for physicians, general practitioners, and related caregiving professions. This can be trickled further down to the public at large with gatekeeper training^60,61^, through which community members from different sectors of society can be better equipped to actively check on, better spot warning signs in, and instill timely hope in, their family and friends.

Lastly, findings from^19^ rightly highlighted a need for suicide research to move beyond conventional methods in order to realize further strides in the field. While machine learning approaches advocated by the authors hold immense potential, e.g., by enabling researchers to simultaneously analyze myriads of factors and their interactions, these approaches typically suffer from the curse of dimensionality, i.e., the amount of data needed to obtain reliable results increases exponentially with the number of variables included. Subsequently, this bottleneck could render machine learning approaches prohibitive to those researchers who may lack access to massive amounts of data. The network approach presented in this paper, therefore, may serve as an alternative, less data-intensive framework to move the needle forward for scholarly discourse in suicide prevention.

Several limitations of this study should nevertheless be noted. First, the study population’s representation warrants caution in generalizing findings arising from this study. Given the poor mental health profile of the study samples and that they were largely recruited through community outreach organizations, they would not correspond to mentally healthy populations. Nevertheless, the sampling procedure suggested that the resulting study population should adequately represent community-dwelling young people (10–24 year-olds) and young adults (25–35 year-olds) in Hong Kong who were concerned about their mental health and well-being. The ethnic composition of the study population could not be ascertained as well, as the surveys did not ask participants’ ethnicity.

The survey design in this study also limited its scope. Survey questions on participants’ past suicidality did not differentiate between the enquired period, i.e., one month prior to taking the survey, and the rest of participants’ lifetimes, thereby preventing participants’ lifetime prevalence of suicidality to be further classified into the history of suicidality and current suicidality. The survey design also precluded testing of different theories of suicide. In order to draw explicit comparisons between 3ST and other prevailing theories, e.g., the Interpersonal Theory of Suicide^22^, future studies may include items measuring perceived burdensomeness and thwarted belongingness.

Importantly, the major limitation of this study would be the skip-structure of SIDAS, whereby participants who never had any suicidal thoughts in the past month, i.e., scored zero in the first item, would ‘skip’ the remaining four items in the scale and score a total of zero. This was reflected in the resulting distribution of SIDAS scores being right-skewed (see Supplementary Fig. 1). While debates in the literature have suggested that skip-structures could result in missing values in the dataset and thereby render it less suitable to be modeled as Gaussian graphical models (e.g., see^62^ for a thorough discussion), we argue that the unanswered SIDAS items due to the skip-structure here should nonetheless not be taken as missing data. This is because SIDAS items relate to frequency at which an individual experiences symptoms and thus the skipped items cannot be treated as missing values; instead, they should be interpreted as “if one does not have suicidal thoughts, one cannot be tormented by suicidal thoughts, have their daily activities interfered by suicidal thoughts, and so on", and therefore should not invalidate the network approach in this study.

Finally, panel network analyses’ results need to be interpreted with caution due to several factors that might have affected the results. Changing contexts of the Hong Kong society between 2018 to 2020—with the social unrest in 2019 and onset of the COVID-19 pandemic in 2020—might confound the panels’ cross-sections (in Supplementary Fig. 8) and consequently might affect the network structures. It should also be noted that the sample size available for estimating the panel networks was small, and this could possibly affect estimates of network structures. In particular, the strong negative edge in the between-subjects network (Fig. 3C) might not have been a real effect and instead might have been due to estimator problems. Furthermore, mismatches between the time gap between panels and survey measurements’ timeframes should warrant caution in interpreting the temporal network’s structure in this study. To tap a fuller extent of the potential of GVAR in panel network analyses, future research should incorporate appropriate mechanisms to attract and retain large sample sizes, and also ensure appropriate match between psychometric scales’ timeframe, time gap between panels, and the time scales in which constructs of interest unfold, as between-person relationships at one time scale may very well be within-person relationships at another time scale^42,46^.

In summary, network analysis in this study suggested that hopelessness might be a key affect in the emergence of suicidal ideation in distressed community-dwelling young people and young adults. Further, it appeared to mediate effects of other well-established risk factors of suicidal ideation such as sleep troubles, anxiety, and poor interpersonal relationships.

Findings here provide an evidence base for both professional training in caregiving professions—including physicians and general practitioners—as well as gatekeeper training in community members to emphasize more on how to effectively recognize hopelessness, and instill hope, in young people and young adults for various types of distress. Timely treatment of hopelessness at the community level may pre-emptively deactivate the pathway to suicidality in distressed individuals, and subsequently serve to reduce the incidence of suicidality at the population level.

### Supplementary information

Supplementary information

Reporting Summary

## Data Availability

The data that support the findings of this study are available from the corresponding author upon reasonable request.

## Code availability

Codes for all analyses conducted in this study can be obtained from the first author upon reasonable request. All codes are written in R. R version 4.2.1^37^ within RStudio^38^ is used in the present study.
