# Supplementary material for: Preventing comorbidity between distress and suicidality: a network analysis
Source: Npj Ment Health Res. 2023 Mar 4;2:2. doi: 10.1038/s44184-023-00022-1 (PMC9984753; doi:10.1038/s44184-023-00022-1)
Supplement: Supplementary file 1 — Supplementary information [file 44184_2023_22_MOESM1_ESM.pdf]

# Supplementary tables

| Please answer the following questions based on how you feel in the past two weeks.<br>Do you recently |
|---|
| 1. Feel headache or pressure in your head? |
| 2. Feel irregular heartbeat or increase in heartbeat and worried that you might have heart problem? |
| 3. Feel discomfort or pressure in your chest? |
| 4. Feel shakiness or numbness in your limbs? |
| 5. Have trouble with sleep? |
| 6. Feel you are carrying too much burden? |
| 7. Feel you are losing confidence in yourself? |
| 8. Feel nervous and tense? |
| 9. Feel worried about your family or friends? |
| 10. Feel that life is entirely hopeless? |
| 11. Feel you get along well with your family or friends? |
| 12. Feel hopeful about the future? |

Supplementary Table 1: CHQ-12 questionnaire used in the surveys. Each question had a Likert-scale response of: 0:“not at all”), 1:“same as usual”), 2:“rather more than usual”), and 3:“much more than usual”). Responses for questions 11 & 12 were first reverse-coded before conducting data analysis.

| Please answer the following questions based on your experiences in the past month.<br>In the past month, | |
|---|---|
| 1. How often have you had thoughts about suicide? | 0:“never” - 10:“always” |
| 2. How much control have you had over these thoughts? | 0:“no control at all” - 10:“full control” |
| 3. How close have you come to making a suicide attempt? | 0:“never” - 10:“have already attempted at least once” |
| 4. To what extent have you felt tormented by thoughts about suicide? | 0:“not at all” - 10:“very much” |
| 5. How much have thoughts about suicide interfered with your ability to carry out daily activities, such as study, work, household tasks or social activities? | 0:“not at all” - 10:“very much” |

Supplementary Table 2: SIDAS questionnaire used in the surveys. Each question had a Likert-scale response from 0 to 10. Responses for question 2 were first reverse-coded before conducting data analysis.

## Supplementary figures

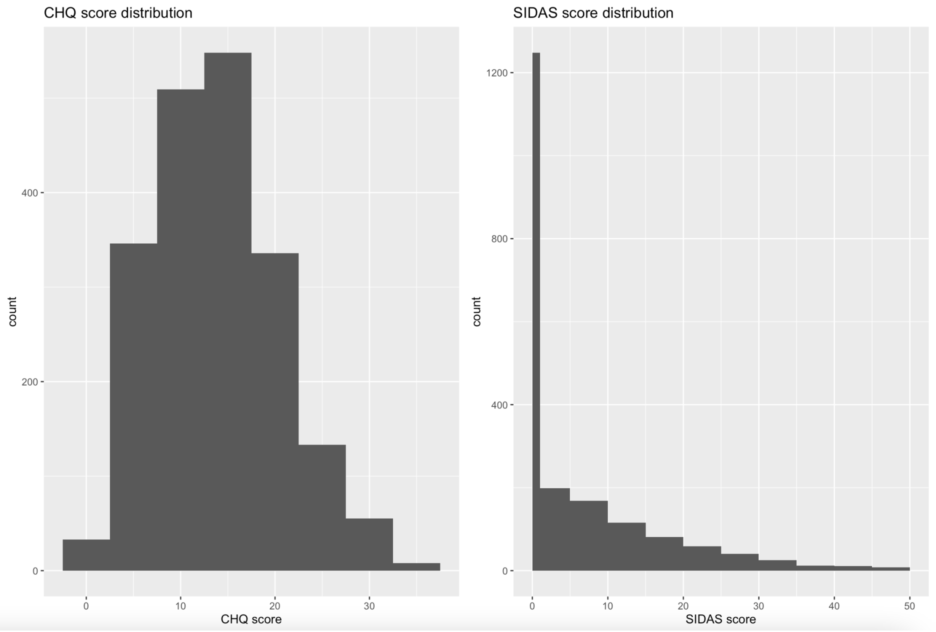


Supplementary Figure 1: Distributions of CHQ and SIDAS responses in the baseline survey. Skewness and kurtosis of the CHQ scores' distribution were 0.48 and 2.85 respectively, indicating a nearly symmetrical distribution that was slightly light-tailed relative to a normal distribution. Skewness and kurtosis of the SIDAS scores' distribution were 2.20 and 7.79 respectively, indicating a right-skewed distribution with a heavier tail than a normal distribution.

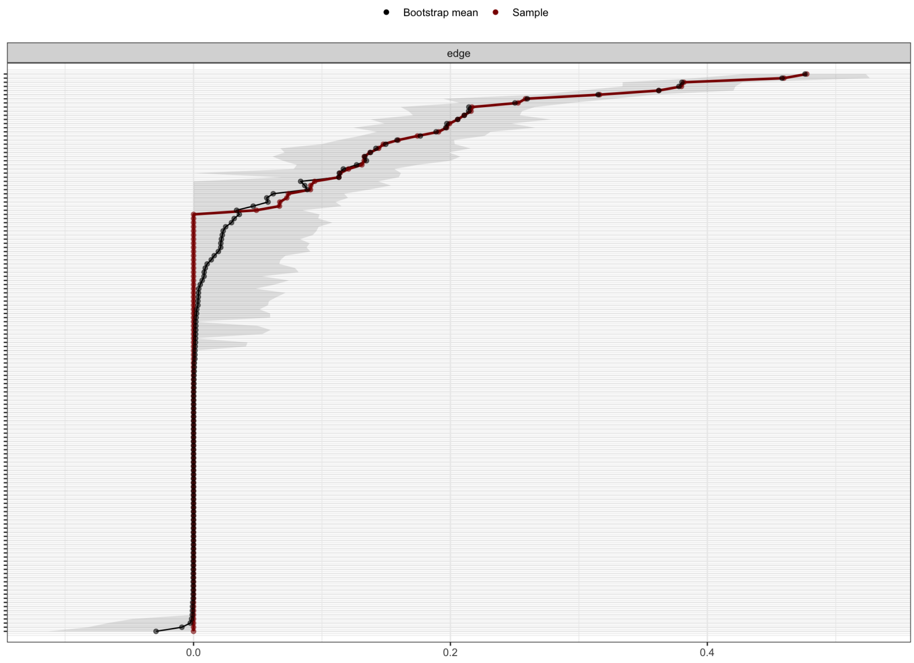


Supplementary Figure 2: Bootstrapped estimates of edge weights in the full-sample symptom network. Horizontal axis represents values of edge weights, while the 272 (17 x 16) ticks at the vertical axis denote all possible distinct edge weights in the symptom network. Red dots denote edge weights in the network realization in Figure 1. Black dots denote bootstrapped means of the respective edge weights. Shaded portions to the left and right of each black dot denote bootstrapped 95% confidence intervals.

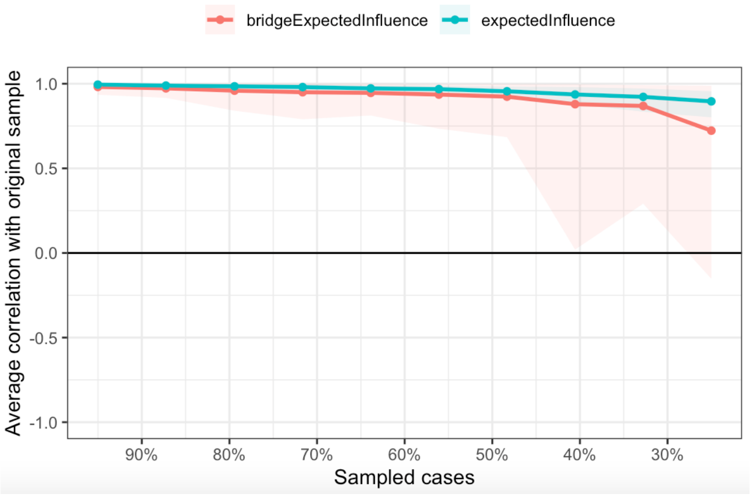


Supplementary Figure 3: Stability of centrality indices in the full-sample symptom network, measured through 1000 case-dropping subset bootstraps. Horizontal axis denotes the percentage of cases / individuals of the study population used. Vertical axis denotes the average of bootstrapped CS-coefficients, i.e., correlations between centrality indices for networks constructed from the study population and those for networks that were re-estimated from the sampled cases only. Red line and blue line represent bootstrapped means of bridge expected influence (EI) and node EI respectively. Red and blue shaded regions represent the bootstrapped 95% confidence intervals of their corresponding indices.

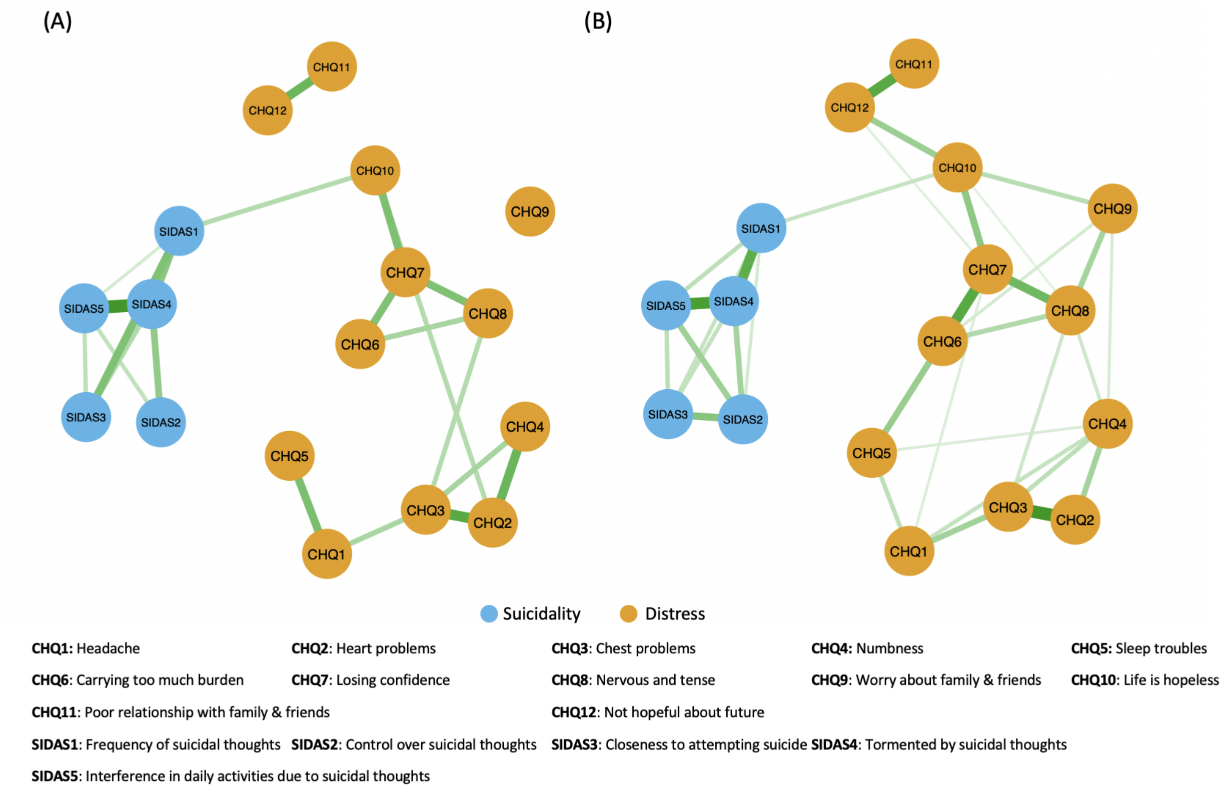


Supplementary Figure 4: Distress-suicidality symptom networks constructed from responses of (A) 195 individuals with, and (B) 1773 individuals without, diagnosis of MDD or schizophrenia in the baseline 2018 survey. Edge thickness represents magnitude of partial correlation between symptoms. Blue nodes and orange nodes represent suicidality and distress symptoms respectively. All edge weights are positive.

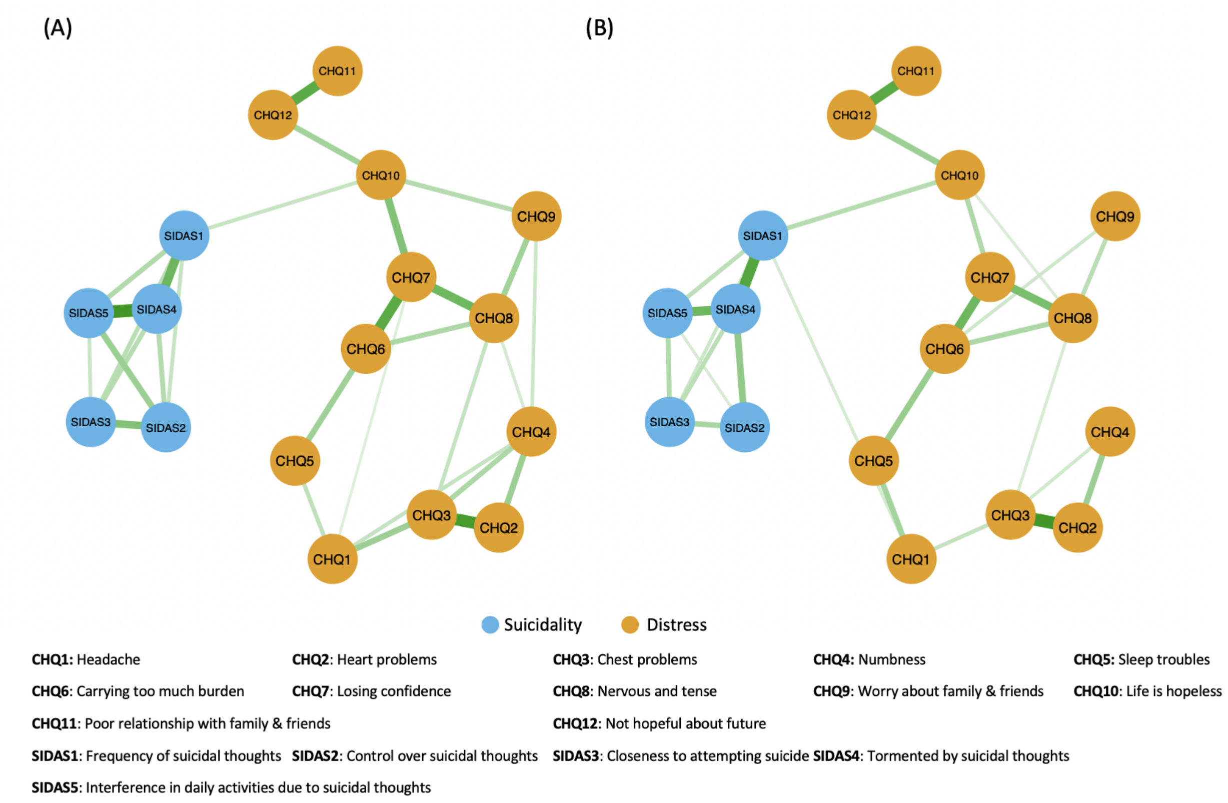


Supplementary Figure 5: Distress-suicidality symptom networks constructed from responses of (A) 1355 young people (10-24 year-old individuals), and (B) 613 young adults (25-35 year-old individuals). Edge thickness represents magnitude of partial correlation between symptoms. Blue nodes and orange nodes represent suicidality and distress symptoms respectively. All edge weights are positive.

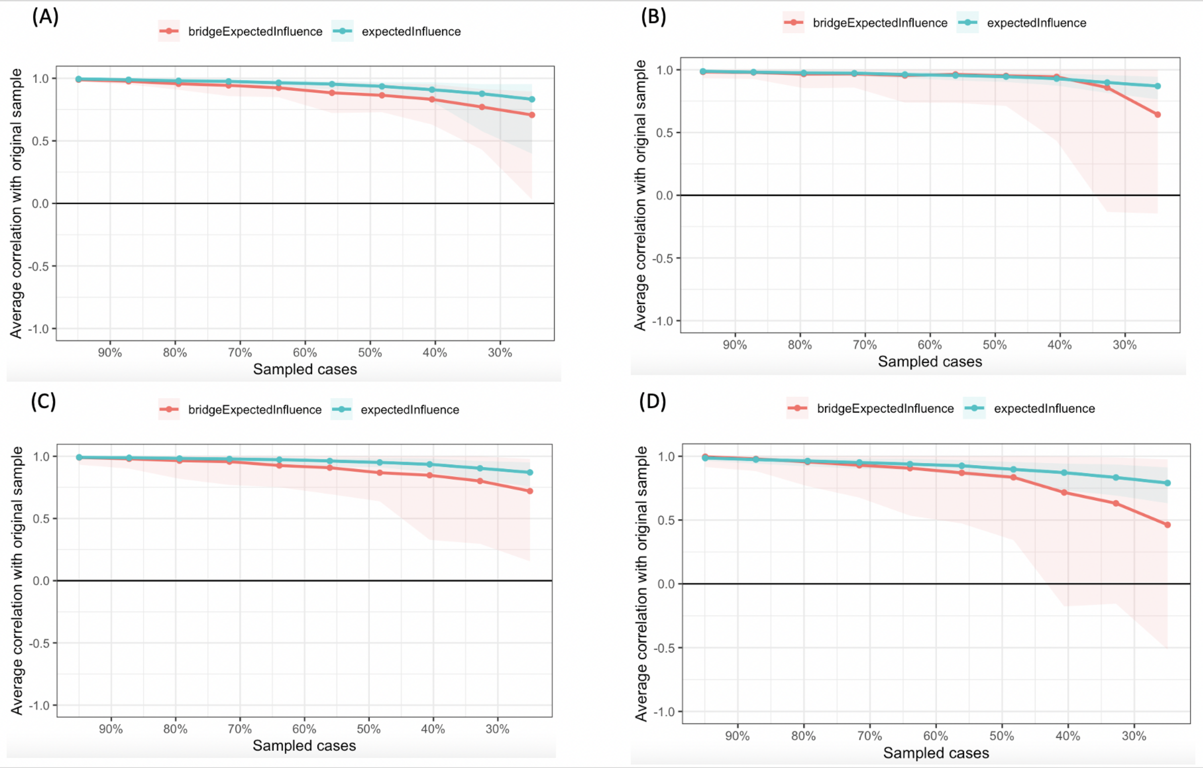


Supplementary Figure 6: Stability of bridge EI and node EI of sub-sample networks for (A) 195 individuals with and (B) 1773 individuals without psychiatric diagnoses; and (C) 1355 young people and (D) 613 young adults. Horizontal axes denote the percentage of cases / individuals of the study population used, and vertical axes denote the average of CS-coefficients calculated from 1000 case-dropping subset bootstraps. CS-coefficients of bridge EI and node EI are (A) 0.52 and 0.60 respectively, (B) 0.44 and 0.75, (C) 0.44 and 0.75, and (D) 0.28 and 0.67 respectively. Red line and blue line represent bootstrapped means of bridge expected influence (EI) and node EI respectively. Red and blue shaded regions represent the bootstrapped 95% confidence intervals of their corresponding indices.

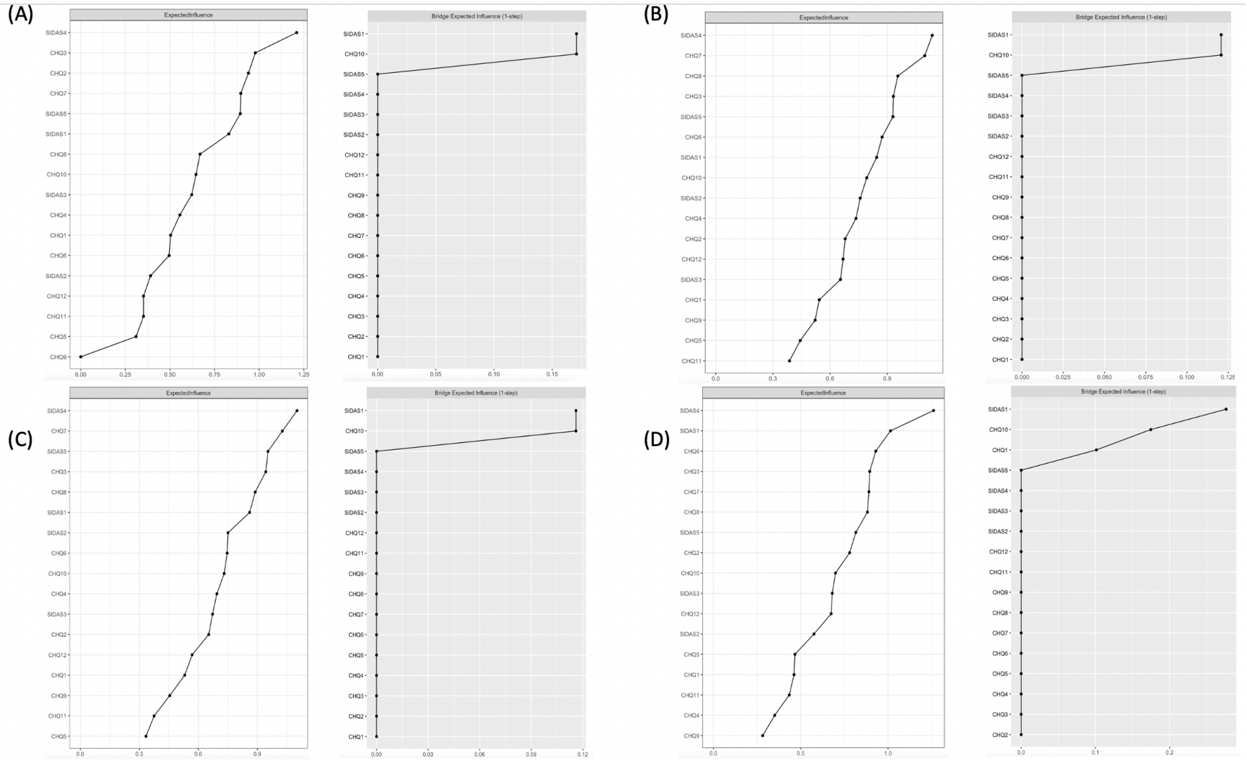


Supplementary Figure 7: Values of each node's bridge EI and node EI in all estimated subsample networks: (A) individuals with, and (B) individuals without, psychiatric diagnoses; and (C) young people and (D) young adults. CHQ1: Headache; CHQ2: Heart problems; CHQ3: Chest problems; CHQ4: Numbness; CHQ5: Sleep troubles; CHQ6: Carrying too much burden; CHQ7: Losing confidence; CHQ8: Nervous and tense; CHQ9: Worry about family & friends; CHQ10: Life is hopeless; CHQ11: Poor relationship with family & friends; CHQ12: Not hopeful about future; SIDAS1: Frequency of suicidal thoughts; SIDAS2: Control over suicidal thoughts; SIDAS3: Closeness to attempting suicide; SIDAS4: Tormented by suicidal thoughts; SIDAS5: Interference in daily activities due to suicidal thoughts.

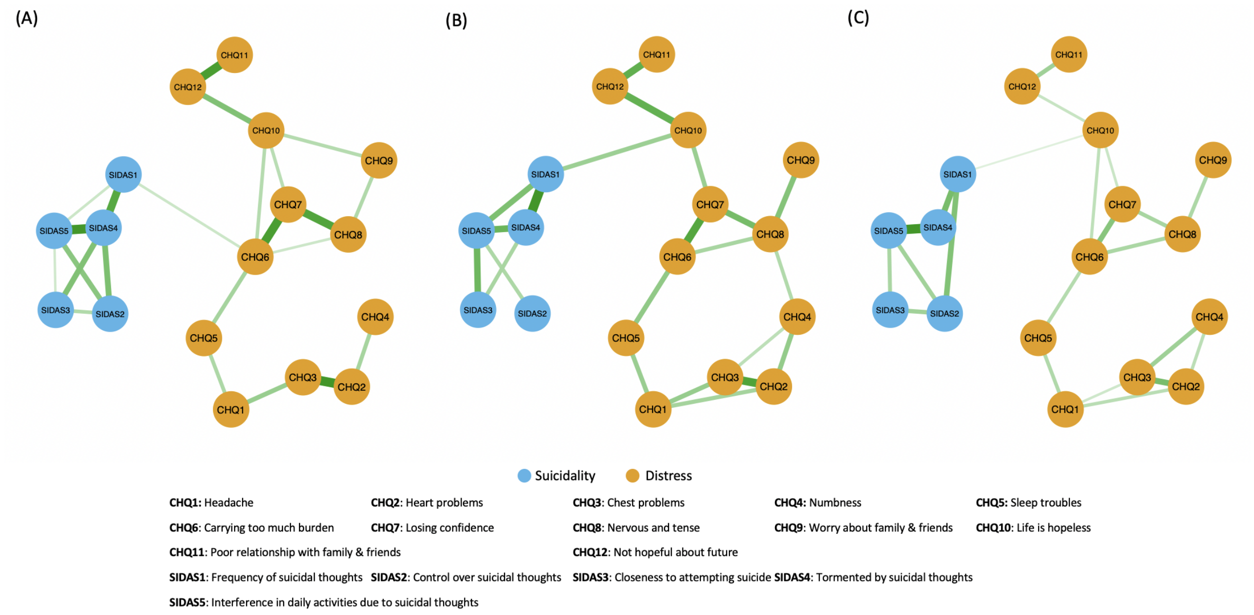


Supplementary Figure 8: Distress-suicidality symptom networks constructed from responses of the same 453 individuals in (A) the 2018 survey, (B) the 2019 survey, and (C) the 2020 survey. Edge thickness represents magnitude of partial correlation between symptoms. Blue nodes and orange nodes represent suicidality and distress symptoms respectively. All edge weights are positive.
